# 5-Ethyl-4-methyl-1*H*-pyrazol-3(2*H*)-one

**DOI:** 10.1107/S160053681001696X

**Published:** 2010-05-15

**Authors:** Tara Shahani, Hoong-Kun Fun, R. Venkat Ragavan, V. Vijayakumar, S. Sarveswari

**Affiliations:** a X-ray Crystallography Unit, School of Physics, Universiti Sains Malaysia, 11800 USM, Penang, Malaysia; b Organic Chemistry Division, School of Advanced Sciences, VIT University, Vellore 632 014, India

## Abstract

In the title compound, C_6_H_10_N_2_O, the 2,3-dihydro-1*H*-pyrazole ring is approximately planar, with a maximum deviation of 0.013 (1) Å. Pairs of inter­molecular N—H⋯O hydrogen bonds link neighboring mol­ecules into dimers, generating *R*_2_^2^(8) ring motifs. These dimers are further linked into two-dimensional arrays parallel to the *bc* plane by inter­molecular N—H⋯O hydrogen bonds. The crystal structure is further stabilized by C—H⋯π inter­actions.

## Related literature

For the background to and the biological activity of 3-ethyl-4-methyl-1*H*-pyrazol-5-ol, see: Brogden (1986 ▶); Coersmeier *et al.* (1986); Gursoy *et al.* (2000 ▶); Ragavan *et al.* (2009 ▶, 2010 ▶); Watanabe *et al.* (1984 ▶); Kawai *et al.* (1997 ▶); Wu *et al.* (2002 ▶). For related structures, see: Shahani *et al.* (2009 ▶, 2010 ▶). For hydrogen-bond motifs, see: Bernstein *et al.* (1995 ▶). For reference bond-length data, see: Allen *et al.* (1987 ▶). For the stability of the temperature controller used for the data collection, see: Cosier & Glazer (1986 ▶).
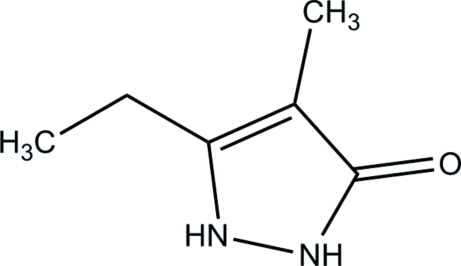


## Experimental

### 

#### Crystal data

C_6_H_10_N_2_O
*M* *_r_* = 126.16
Monoclinic, 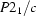

*a* = 8.374 (2) Å
*b* = 7.2881 (16) Å
*c* = 11.300 (3) Å
β = 109.955 (5)°
*V* = 648.3 (3) Å^3^
*Z* = 4
Mo *K*α radiation
μ = 0.09 mm^−1^
*T* = 100 K
0.52 × 0.16 × 0.09 mm

#### Data collection

Bruker APEXII DUO CCD area-detector diffractometer
Absorption correction: multi-scan (*SADABS*; Bruker, 2009 ▶) *T*_min_ = 0.954, *T*_max_ = 0.992
10018 measured reflections
2745 independent reflections
2325 reflections with *I* > 2σ(*I*)
*R*_int_ = 0.029

#### Refinement

*R*[*F* ^2^ > 2σ(*F* ^2^)] = 0.039
*wR*(*F* ^2^) = 0.123
*S* = 1.14
2745 reflections
122 parameters
All H-atom parameters refined
Δρ_max_ = 0.52 e Å^−3^
Δρ_min_ = −0.35 e Å^−3^

### 

Data collection: *APEX2* (Bruker, 2009 ▶); cell refinement: *SAINT* (Bruker, 2009 ▶); data reduction: *SAINT*; program(s) used to solve structure: *SHELXTL* (Sheldrick, 2008 ▶); program(s) used to refine structure: *SHELXTL*; molecular graphics: *SHELXTL*; software used to prepare material for publication: *SHELXTL* and *PLATON* (Spek, 2009 ▶).

## Supplementary Material

Crystal structure: contains datablocks global, I. DOI: 10.1107/S160053681001696X/wn2385sup1.cif

Structure factors: contains datablocks I. DOI: 10.1107/S160053681001696X/wn2385Isup2.hkl

Additional supplementary materials:  crystallographic information; 3D view; checkCIF report

## Figures and Tables

**Table 1:** Hydrogen-bond geometry (Å, °) *Cg*1 is the centroid of the 1*H*-pyrazole ring (C1–C3/N1/N2).

*D*—H⋯*A*	*D*—H	H⋯*A*	*D*⋯*A*	*D*—H⋯*A*
N1—H1*N*1⋯O1^i^	0.902 (15)	1.829 (15)	2.7267 (11)	174.0 (16)
N2—H1*N*2⋯O1^ii^	0.972 (14)	1.715 (14)	2.6777 (10)	169.9 (13)
C5—H5*A*⋯*Cg*1^iii^	1.013 (13)	2.896 (15)	3.6749 (14)	134.2 (11)

Symmetry codes: (i) 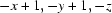; (ii) 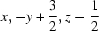; (iii) 
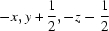
.

**Fig. 1.**
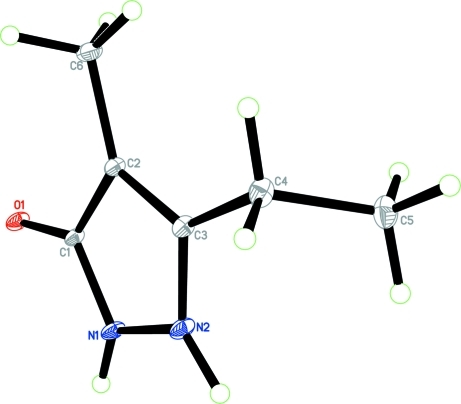
The molecular structure of the title compound, showing 30% probability displacement ellipsoids and the atom numbering scheme. Hydrogen atoms are shown as spheres of arbitrary radius.

**Fig. 2.**
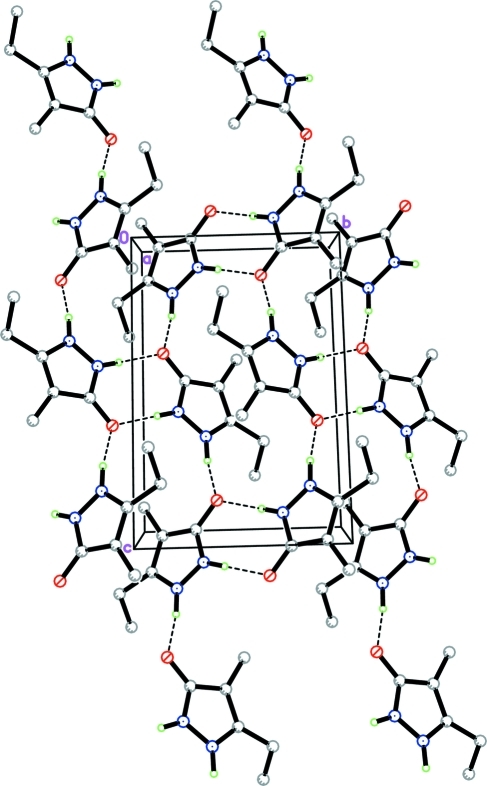
The crystal packing of the title compound, showing a 2D array parallel to the bc plane. Hydrogen bonds are denoted by dashed lines. H atoms not involved in the hydrogen bond interactions have been omitted for clarity.

## supplementary crystallographic information

### Comment

Pyrazolone derivatives have a broad spectrum of biological activities as analgesic, antipyretic and anti-inflammatory therapeutical drugs (Brogden, 1986; Gursoy *et al.*, 2000). A class of new pyrazolone compounds have been synthesized and reported to exhibit antibacterial and antifungal activities (Ragavan *et al.*, 2010; Ragavan *et al.*, 2009). A new pyrazolone derivative, edaravone (5-ethyl-4-methyl-1*H*-pyrazol-3(2*H*)-one), is being used as a drug in clinical practice for brain ischemia (Watanabe *et al.*, 1984; Kawai *et al.*, 1997) and it has also been found to be effective against myocardial ischemia (Wu *et al.*, 2002).

In the crystal structure (Fig. 1), the 2,3-dihydro-1*H*-pyrazole ring (C1–C3/N1/N2) is approximately planar with a maximum deviation of 0.013 (1) Å for atoms N1 and N2 (but they are on opposite sides of the plane). The bond lengths (Allen *et al.*, 1987) and angles are within normal ranges and comparable to those in closely related structures reported recently (Shahani *et al.*, 2009; 2010).

In the crystal packing (Fig. 2), pairs of intermolecular N1—H1N1···O1 hydrogen bonds (Table 1) link neighboring molecules into dimers, generating *R*^2^_2_(8) ring motifs (Bernstein *et al.*, 1995). These dimers are further linked into 2D arrays parallel to the *bc* plane by intermolecular N2—H1N2···O1 hydrogen bonds (Table 1). The crystal structure is further stabilized by a C—H···π interaction (Table 1), involving the C1–C3/N1/N2 ring (centroid *Cg*1) .

### Experimental

The compound 5-ethyl-4-methyl-1*H*-pyrazol-3(2*H*)-one has been synthesized using the method reported in the literature (Ragavan *et al.*, 2009, 2010) and purified by column chromatography (MeOH: EtOAc, 1:99). It was recrystallised as a colourless solid, using ethanol. *Mp*: 496.4–507.1 K; MS calculated for C_6_H_10_N_2_O: 126.15. Found: 128.0 (M+).

### Refinement

All hydrogen atoms were located in a difference map and were refined freely [N–H = 0.902 (14) – 0.972 (14) Å; C–H = 0.989 (13) – 1.015 (13) Å].

### Crystal data

**Table:**

C_6_H_10_N_2_O	*F*(000) = 272
*M**_r_* = 126.16	*D*_x_ = 1.293 Mg m^−^^3^
Monoclinic, *P*2_1_/*c*	Mo *K*α radiation, λ = 0.71073 Å
Hall symbol: -P 2ybc	Cell parameters from 3666 reflections
*a* = 8.374 (2) Å	θ = 2.6–34.5°
*b* = 7.2881 (16) Å	µ = 0.09 mm^−^^1^
*c* = 11.300 (3) Å	*T* = 100 K
β = 109.955 (5)°	Plate, colourless
*V* = 648.3 (3) Å^3^	0.52 × 0.16 × 0.09 mm
*Z* = 4	

### Data collection

**Table:**

Bruker APEXII DUO CCD area-detector diffractometer	2745 independent reflections
Radiation source: fine-focus sealed tube	2325 reflections with *I* > 2σ(*I*)
graphite	*R*_int_ = 0.029
φ and ω scans	θ_max_ = 34.6°, θ_min_ = 3.4°
Absorption correction: multi-scan (*SADABS*; Bruker, 2009)	*h* = −13→13
*T*_min_ = 0.954, *T*_max_ = 0.992	*k* = −11→11
10018 measured reflections	*l* = −18→17

### Refinement

**Table:**

Refinement on *F*^2^	Primary atom site location: structure-invariant direct methods
Least-squares matrix: full	Secondary atom site location: difference Fourier map
*R*[*F*^2^ > 2σ(*F*^2^)] = 0.039	Hydrogen site location: inferred from neighbouring sites
*wR*(*F*^2^) = 0.123	All H-atom parameters refined
*S* = 1.14	*w* = 1/[σ^2^(*F*_o_^2^) + (0.0715*P*)^2^ + 0.0472*P*] where *P* = (*F*_o_^2^ + 2*F*_c_^2^)/3
2745 reflections	(Δ/σ)_max_ < 0.001
122 parameters	Δρ_max_ = 0.52 e Å^−^^3^
0 restraints	Δρ_min_ = −0.35 e Å^−^^3^

### Special details

**Table:**

Experimental. The crystal was placed in the cold stream of an Oxford Cyrosystems Cobra open-flow nitrogen cryostat (Cosier & Glazer, 1986) operating at 100.0 (1) K.
Geometry. All esds (except the esd in the dihedral angle between two l.s. planes) are estimated using the full covariance matrix. The cell esds are taken into account individually in the estimation of esds in distances, angles and torsion angles; correlations between esds in cell parameters are only used when they are defined by crystal symmetry. An approximate (isotropic) treatment of cell esds is used for estimating esds involving l.s. planes.
Refinement. Refinement of *F*^2^ against ALL reflections. The weighted *R*-factor wR and goodness of fit *S* are based on *F*^2^, conventional *R*-factors *R* are based on *F*, with *F* set to zero for negative *F*^2^. The threshold expression of *F*^2^ > σ(*F*^2^) is used only for calculating *R*-factors(gt) etc. and is not relevant to the choice of reflections for refinement. *R*-factors based on *F*^2^ are statistically about twice as large as those based on *F*, and *R*- factors based on ALL data will be even larger.

### Fractional atomic coordinates and isotropic or equivalent isotropic displacement parameters (Å^2^)

**Table:**

	*x*	*y*	*z*	*U*_iso_*/*U*_eq_	
O1	0.42822 (7)	0.62337 (8)	0.11992 (5)	0.01463 (13)	
N1	0.42529 (8)	0.69441 (9)	−0.08076 (6)	0.01353 (13)	
N2	0.35794 (9)	0.82809 (9)	−0.16813 (6)	0.01431 (13)	
C1	0.38533 (9)	0.73007 (10)	0.02374 (6)	0.01110 (13)	
C2	0.29351 (9)	0.89787 (9)	0.00188 (6)	0.01142 (13)	
C3	0.28136 (9)	0.95309 (10)	−0.11791 (7)	0.01249 (14)	
C4	0.19811 (10)	1.11714 (10)	−0.19250 (7)	0.01638 (15)	
C5	0.05452 (11)	1.06785 (12)	−0.31308 (8)	0.02089 (17)	
C6	0.22769 (10)	0.99011 (11)	0.09370 (7)	0.01700 (15)	
H4A	0.1538 (18)	1.1950 (18)	−0.1386 (13)	0.026 (3)*	
H4B	0.2822 (16)	1.1904 (17)	−0.2159 (11)	0.019 (3)*	
H5A	−0.0064 (17)	1.1779 (18)	−0.3632 (13)	0.025 (3)*	
H5B	−0.0336 (19)	0.991 (2)	−0.2946 (14)	0.038 (4)*	
H5C	0.0961 (19)	0.994 (2)	−0.3704 (15)	0.036 (4)*	
H6A	0.3195 (17)	1.0187 (18)	0.1773 (13)	0.027 (3)*	
H6B	0.147 (2)	0.9115 (19)	0.1185 (14)	0.033 (4)*	
H6C	0.163 (2)	1.103 (2)	0.0557 (16)	0.044 (4)*	
H1N1	0.4808 (19)	0.5936 (19)	−0.0921 (14)	0.028 (3)*	
H1N2	0.3762 (17)	0.8332 (19)	−0.2486 (13)	0.028 (3)*	

### Atomic displacement parameters (Å^2^)

**Table:**

	*U*^11^	*U*^22^	*U*^33^	*U*^12^	*U*^13^	*U*^23^
O1	0.0221 (3)	0.0147 (2)	0.0089 (2)	0.00498 (18)	0.00754 (19)	0.00296 (17)
N1	0.0211 (3)	0.0125 (3)	0.0092 (2)	0.0048 (2)	0.0080 (2)	0.00233 (19)
N2	0.0221 (3)	0.0130 (3)	0.0098 (3)	0.0033 (2)	0.0080 (2)	0.0027 (2)
C1	0.0144 (3)	0.0120 (3)	0.0078 (3)	0.0006 (2)	0.0050 (2)	−0.0002 (2)
C2	0.0142 (3)	0.0109 (3)	0.0096 (3)	0.0009 (2)	0.0047 (2)	−0.0003 (2)
C3	0.0159 (3)	0.0106 (3)	0.0110 (3)	0.0000 (2)	0.0047 (2)	0.0000 (2)
C4	0.0213 (3)	0.0118 (3)	0.0143 (3)	0.0012 (2)	0.0039 (3)	0.0027 (2)
C5	0.0212 (3)	0.0193 (3)	0.0178 (3)	0.0024 (3)	0.0010 (3)	0.0032 (3)
C6	0.0208 (3)	0.0187 (3)	0.0132 (3)	0.0048 (3)	0.0079 (3)	−0.0015 (3)

### Geometric parameters (Å, °)

**Table:**

O1—C1	1.2839 (9)	C4—C5	1.5209 (12)
N1—C1	1.3578 (9)	C4—H4A	0.993 (14)
N1—N2	1.3645 (9)	C4—H4B	0.989 (13)
N1—H1N1	0.902 (14)	C5—H5A	1.013 (13)
N2—C3	1.3459 (10)	C5—H5B	1.003 (15)
N2—H1N2	0.972 (14)	C5—H5C	0.992 (16)
C1—C2	1.4206 (10)	C6—H6A	1.015 (13)
C2—C3	1.3823 (10)	C6—H6B	0.994 (15)
C2—C6	1.4908 (10)	C6—H6C	1.000 (16)
C3—C4	1.4916 (11)		
			
C1—N1—N2	109.19 (6)	C5—C4—H4A	109.8 (8)
C1—N1—H1N1	124.9 (9)	C3—C4—H4B	110.2 (7)
N2—N1—H1N1	125.8 (9)	C5—C4—H4B	107.8 (7)
C3—N2—N1	108.49 (6)	H4A—C4—H4B	107.7 (11)
C3—N2—H1N2	128.1 (8)	C4—C5—H5A	114.0 (8)
N1—N2—H1N2	123.1 (8)	C4—C5—H5B	111.0 (9)
O1—C1—N1	122.64 (7)	H5A—C5—H5B	106.9 (12)
O1—C1—C2	130.32 (6)	C4—C5—H5C	111.5 (9)
N1—C1—C2	107.04 (6)	H5A—C5—H5C	106.6 (12)
C3—C2—C1	105.99 (6)	H5B—C5—H5C	106.3 (12)
C3—C2—C6	128.98 (7)	C2—C6—H6A	113.4 (8)
C1—C2—C6	125.03 (6)	C2—C6—H6B	112.5 (9)
N2—C3—C2	109.23 (6)	H6A—C6—H6B	103.1 (11)
N2—C3—C4	120.16 (7)	C2—C6—H6C	110.4 (10)
C2—C3—C4	130.59 (7)	H6A—C6—H6C	110.9 (12)
C3—C4—C5	113.02 (7)	H6B—C6—H6C	106.1 (13)
C3—C4—H4A	108.2 (8)		
			
C1—N1—N2—C3	2.59 (8)	N1—N2—C3—C4	179.19 (6)
N2—N1—C1—O1	177.88 (7)	C1—C2—C3—N2	0.73 (8)
N2—N1—C1—C2	−2.09 (8)	C6—C2—C3—N2	−179.69 (7)
O1—C1—C2—C3	−179.13 (7)	C1—C2—C3—C4	179.34 (7)
N1—C1—C2—C3	0.84 (8)	C6—C2—C3—C4	−1.08 (13)
O1—C1—C2—C6	1.27 (12)	N2—C3—C4—C5	60.72 (10)
N1—C1—C2—C6	−178.76 (7)	C2—C3—C4—C5	−117.76 (9)
N1—N2—C3—C2	−2.03 (8)		

### Hydrogen-bond geometry (Å, °)

**Table:**

Cg1 is the centroid of the 1*H*-pyrazole ring (C1–C3/N1/N2).

**Table:**

*D*—H···*A*	*D*—H	H···*A*	*D*···*A*	*D*—H···*A*
N1—H1N1···O1^i^	0.902 (15)	1.829 (15)	2.7267 (11)	174.0 (16)
N2—H1N2···O1^ii^	0.972 (14)	1.715 (14)	2.6777 (10)	169.9 (13)
C5—H5A···Cg1^iii^	1.013 (13)	2.896 (15)	3.6749 (14)	134.2 (11)

Symmetry codes: (i) −*x*+1, −*y*+1, −*z*; (ii) *x*, −*y*+3/2, *z*−1/2; (iii) −*x*, *y*+1/2, −*z*−1/2.
